# Adaptive Color Calibration Based One-Shot Structured Light System

**DOI:** 10.3390/s120810947

**Published:** 2012-08-08

**Authors:** Yu Zhou, Dongwei Zhao, Yao Yu, Jie Yuan, Sidan Du

**Affiliations:** School of Electronic Science and Engineering, Nanjing University, Nanjing 210093, Jiangsu, China; E-Mails: zhaodw320@sina.com (D.Z.); allanyu@nju.edu.cn (Y.Y.); yuanjie@nju.edu.cn (J.Y.)

**Keywords:** 3D image acquisition, adaptive color calibration, discrete trend transform, structured light

## Abstract

In one-shot color structured light systems, the color of stripe patterns are typically distorted with respect to color crosstalk, ambient light and the albedo of the scanned objects, leading to mismatch in the correspondence of color stripes between the projected and captured images. In this paper, an adaptive color calibration and Discrete Trend Transform algorithm are presented to achieve high-resolution 3D reconstructions. The adaptive color calibration, according to the relative albedo in RGB channels, can improve the accuracy of labeling stripe by alleviating the effect of albedo and ambient light while decoding the color. Furthermore, the Discrete Trend Transform in the M channel makes the color calibration an effective method for detecting weak stripes due to the uneven surfaces or reflectance characteristics of the scanned objects. With this approach, the presented system is suitable for scanning moving objects and generating high-resolution 3D reconstructions without the need of dark laboratory environments.

## 1. Introduction

3D shape acquisition has received considerable research interest in the last decade. There are some groups of techniques for 3D shape acquisition, such as stereo vision, Time Of Flight (TOF) and structured light [1]. Compared with TOF and stereo vision techniques, the main advantages of structured light techniques include the easy image processing involved and the high accuracy achieved in the 3D reconstruction [2]. Structured light techniques are based on the triangle principle. First, some designed patterns are projected onto the scanned objects, and a camera captures the scene. Second, the correspondence between the projected and captured patterns is established. Finally, the 3D coordinates of the objects are derived from correspondence between the projected and detected patterns that combines the geometric calibration parameters of the projector and camera.

In the development of structured light techniques, the time-multiplexing techniques, also called multi-shots techniques, were the first to appear. These techniques can achieve a high level of accuracy and resolution through encoded points by projecting a sequence of patterns. However, there is a trade-off between the scanning speed and the resolution. In the recent years, fast time-multiplexing techniques have been developed that allow the shape of the moving object to be acquired. However such techniques require specialized hardware. To achieve a high resolution and high speed at the same time, the authors in [3,4] propose a one-shot structured light system for rapid range acquisition. For one-shot structured light systems, the final reconstruction result is significantly affected by three vital processes: the designation of projected patterns, the detection of coded cells in the captured image and the establishment of correspondence between the projected and captured coded cells. In recent years, as more research and efforts have been made in the field, these techniques have generated higher-quality 3D reconstruction results, making the techniques quite useful and widespread in industry and cultural heritage.

The projected pattern has a strong influence on 3D shape acquisition. Clearly, the pattern itself should be easy to detect, and it should be possible to establish correspondence between the projected and captured coded cells. Different patterns can be found in [5,6], including the use of color stripe [7], a unique shape [8], modulated patterns [9] or color-coded grids [10]. In [11], a 2D M-array pattern with a Hamming distance is suggested for robustness consideration. The matrix is comprised of many symbols, and this technique enhances the tolerance of failure to recover symbols. In this pattern, each symbol contains several pixels; thus, the reconstruction resolution is limited. In [12], the authors propose a self-adaptive system for real-time range acquisition that uses pattern color, geometry, tracking and graph cut to solve the corresponding problem. The system using coded colors can obtain denser results than that using shapes or modulated patterns. However, certain factors, such as the reflectance characteristics of the scanned object, ambiguity due to uneven surfaces and ambient light, have a significant effect on color classification and the detection of coded cells. To avoid the influence of these factors, Zhang *et al.* [13] present a method using multi-pass dynamic programming and edge-based reconstruction. This method alleviates albedo influence, but edge-based reconstruction cannot locate the accurate sub-pixel position of the edge. Pages *et al.* [2] propose a peak-based coding strategy that can improve the resolution without loss of accuracy; however, the albedo of the illuminated object is modeled by a static matrix, which limits the application of the method in a dynamic scene. For the purpose of robustness under ambient light, Benveniste *et al.* [14] develop a structured light system that decodes the color using a color invariant and optimizes the projected patterns by flexibly changing stripe color for different colored objects.

Addressing the impacts of these factors, in this paper, we focus on a one-shot color structured light system and propose an adaptive color calibration and Discrete Trend Transform (DTT) algorithm to obtain high-resolution 3D point clouds without the need of dark laboratory environments. First, a relative albedo function between two color channels is proposed to calibrate color adaptively. With this method, distorted color caused by different albedos in RGB channels is calibrated adaptively, and white ambient light in the scene can be canceled during color classification. Second, considering the different intensity caused by uneven surfaces or viewpoints, a novel M channel, DTT and sub-pixel peak localization algorithm are proposed to segment and detect the stripes. These techniques significantly improve the accuracy of locating and labeling the stripes. In the literature, the goal of Fechteler's work is most similar to our work, where *k*-means and clustering are utilized to classify the color adaptively [15].

## 2. System Setup and Framework

To realize high-resolution 3D reconstruction, a DLP projector with a WUXGA resolution (1,920 × 1,080) is used to project structured color light, and a Nikon D200 camera with a resolution of 5.2 mega pixels (2,560 × 2,048) is used to grab the scene. The higher resolutions of the camera and projector allow us to achieve denser and more accurate point clouds. The relative direction angle between the projector and camera is 17°.

To acquire 3D shapes in a natural instead of dark environment, the following steps are performed:

1. Project stripe patterns onto the surface of the target objects and capture an image under ambient light.
2. Calibrate the geometric distortion of the camera and projector. We refer to the method proposed by Pages [2].
3. Adjust the color by static and adaptive color calibration. The color crosstalk is approximated in static color calibration, and then the adaptive color calibration is adopted to adjust color with respect to the object's color and ambient light.
4. Extract the stripes and locate the peaks of the stripes. The proposed DTT algorithm is applied to segment the stripes. Furthermore, the sub-pixel accurate localizations of the peaks are derived.
5. Classify the colors of the stripes and determine the correspondence between the projected and captured patterns by the dynamic programming algorithm [16] with a cost function of the hue value.
6. Generate the 3D point clouds and reconstruct the surfaces of the objects through 3D delaunay triangulation.

### 2.1. Projected Coding Patterns

**Figure 1.**
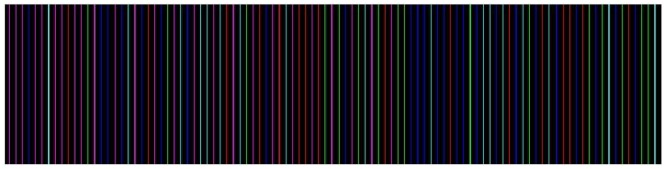
The cut-out of generated stripe patterns.

We use a modified De Bruijn sequence as the arrangement of color stripes to design the projected patterns. Suppose that *D*(*k, n*) denotes a k-array, n-order De Bruijn sequence; then, the element would be *E* = {0, 1, 2, 3, 4, 5}. Each element is assigned a color with a distance of 60° in hue value, as listed in Table 1. We consider that a neighbor stripe should differ by at least two channels in (*R, G, B*). With this constraint, only three nodes are allowed to follow one certain node. For element 0, the possible next following node should only be 1, 2 or 3. In this manner, a De Bruijn sequence with a length of 162 is generated. Figure 1 is the cut-out of the generated stripe patterns.

## 3. Static and Adaptive Color Calibration

The color structured light system in a robust manner consists of two key processes: the recovery of color in the grabbed image in contrast with the projected color on the object's surfaces and the accurate establishment of correspondence between the projected and captured patterns. The color is distorted because of the following reasons:

- The color crosstalk between the projector and camera.
- The ambient light in the environment.
- The different colored object results in different albedos of the RGB channels.
- The stripes in the captured image typically vary in both amplitude and width due to the uneven surfaces or reflectance characteristics of the scanned objects.

### 3.1. Static Color Calibration

The color distortion caused by projector-camera color crosstalk is adjusted by a static color calibration, in which the parameters are stable for a pairwise projector-camera and measured in advance. The camera captures the reflected light through RGB channel sensors as an image. The model of this process was formulated as Equation (1) by Caspi *et al.* [17].

(1) $$\underset{{\text{I}}_{\text{c}}}{\underset{︸}{\left[\begin{array}{c} {\text{c}}^{\text{r}}\\ {\text{c}}^{\text{g}}\\ {\text{c}}^{\text{b}} \end{array}\right]}}=\underset{\text{X}}{\underset{︸}{\left[\begin{array}{ccc} {\text{x}}_{\text{rr}} & {\text{x}}_{\text{rg}} & {\text{x}}_{\text{rb}}\\ {\text{x}}_{\text{gr}} & {\text{x}}_{\text{gg}} & {\text{x}}_{\text{gb}}\\ {\text{x}}_{\text{br}} & {\text{x}}_{\text{bg}} & {\text{x}}_{\text{bb}} \end{array}\right]}}\underset{\text{A}}{\underset{︸}{\left[\begin{array}{ccc} {\text{a}}_{\text{r}} & 0 & 0\\ 0 & {\text{a}}^{\text{g}} & 0\\ 0 & 0 & {\text{a}}_{\text{b}} \end{array}\right]}}(\underset{{\text{I}}_{\text{P}}}{\underset{︸}{\left[\begin{array}{c} {\text{p}}^{\text{r}}\\ {\text{p}}^{\text{g}}\\ {\text{p}}^{\text{b}} \end{array}\right]}}+\underset{\text{O}}{\underset{︸}{\left[\begin{array}{c} {\text{o}}^{\text{r}}\\ {\text{o}}^{\text{g}}\\ {\text{o}}^{\text{b}} \end{array}\right]}})$$

where *I_c_* is the observed color through the camera, and *I_p_* denotes the corresponding projected color projected by the projector. *O* addresses the ambient light illumination. *X* indicates the projector-camera color crosstalk matrix, and *A* represents the albedo matrix of the object surface. During the static calibration process, the color crosstalk matrix *X* in Equation (1) is approximated by projecting solid color stripe patterns to a white planar board.

### 3.2. Adaptive Color Calibration

To recover the stripe's color with respect to the different albedos in the RGB channels and ambient light in the scene, the relative albedo is defined to calibrate the stripe color adaptively, as shown in Equation (2).

(2) $$\text{A}=\left[\begin{array}{ccc} {\text{a}}^{\text{r}} & 0 & 0\\ 0 & \alpha {\text{a}}^{\text{r}} & 0\\ 0 & 0 & \beta {\text{a}}^{\text{r}} \end{array}\right]={\text{a}}^{\text{r}}\left[\begin{array}{ccc} 1 & 0 & 0\\ 0 & \alpha & 0\\ 0 & 0 & \beta \end{array}\right]$$

where *a^r^* is the red channel reflectance ratio, *α* indicates the green channel relative reflectance ratio compared to the red channel, and *β* denotes the blue channel relative reflectance ratio.

In the stripe segmentation and color classification processes, *I_p_* should be used as an input. Unfortunately, *I_p_* is distorted by the crosstalk effect and albedo matrix. Given camera output *I_c_*, we can compute the calibrated color through color calibration, which can be expressed as Equation (3).

(3) $$I_{p}+O={\tilde{A}}^{-1}X^{-1}I_{c}=a^{-r}{\left[\begin{array}{ccc} 1 & 0 & 0\\ 0 & \tilde{\alpha } & 0\\ 0 & 0 & \tilde{\beta } \end{array}\right]}^{-1}X^{-1}I_{c}$$

where *Ã*, compromised with *α̃* and *β̃*, is the estimation of *A*. Then, the calibrated color *I_a_*, defined Equation (4), is the input for stripe segmentation and color classification.

(4) $${\text{I}}_{\text{a}}={\text{a}}^{\text{r}}({\text{I}}_{\text{p}}+\text{O})={\left[\begin{array}{ccc} 1 & 0 & 0\\ 0 & \tilde{\alpha } & 0\\ 0 & 0 & \tilde{\beta } \end{array}\right]}^{-1}{\text{X}}^{-1}{\text{I}}_{c}$$

The color crosstalk matrix *X* can be obtained from static color calibration. In the following section, we conclude that *α̃* and *β̃* can be estimated from the relative albedo estimation. The influence of ambient light *O* and *a^r^* can both be canceled in the color classification and stripe segmentation processes.

### 3.3. Relative Albedo Estimation

We estimate *α̃* by defining a relative albedo function between the red and green channels. Similarly, *β̃* is estimated by comparing the red and blue channels. A histogram for each channel is produced. For example, the histogram of the red channel is expressed as Equation (5):

(5) $${\text{H}}^{\text{r}}=\{({\text{H}}_{1}^{\text{r}},{\text{W}}_{1}^{\text{r}}),({\text{H}}_{2}^{\text{r}},{\text{W}}_{2}^{\text{r}}),\cdots ,({\text{H}}_{\text{n}}^{\text{r}},{\text{W}}_{\text{n}}^{\text{r}})\}$$

where ${\text{H}}_{\text{i}}^{\text{r}}(1\leq \text{i}\leq \text{n})$ is the histogram bin, and ${\text{W}}_{\text{i}}^{\text{r}}(1\leq \text{i}\leq \text{n})$ is the bin value. The superscripts r, g and b denote the red, green and blue channels respectively. To match *H^r^, H^g^* is transformed into *H^g^*′, and a flow matrix **f** = {*f_ij_*} (1 ≤ *i* ≤ *n*, 1 ≤ *j* ≤ *n*) is defined to represent the transition process. A specified **f** minimizes the overall cost function. The cost function is expressed as Equation (6).

(6) $$\text{cost}({\text{H}}^{\text{g}},{\text{H}}^{\text{r}})=\sum_{\text{i}=1}^{\text{n}}\sum_{\text{j}=1}^{\text{n}}|\text{j}-\text{i}|{\text{f}}_{\text{ij}}$$

where *f_ij_* denotes the flow from ${\text{H}}_{\text{i}}^{\text{g}}$ to ${\text{H}}_{\text{j}}^{\text{r}}$ and |*j* − *i*| denotes the flow distance, which implies that we encourage a flow from one bin in the histogram to another bin with a shorter distance. Equations (7)–(10) are the constraints of the process.

(7) $$f_{ij}\geq 0\;\text{for}\;1\leq i\leq n,1\leq j\leq n$$

(8) $$\sum_{\text{i}=1}^{\text{n}}{\text{f}}_{\text{ij}}\leq {\text{W}}_{\text{j}}^{\text{r}},1\leq \text{j}\leq \text{n}$$

(9) $$\sum_{\text{j}=1}^{\text{n}}{\text{f}}_{\text{ij}}\leq {\text{W}}_{\text{i}}^{\text{g}},1\leq \text{j}\leq \text{n}$$

(10) $$\sum_{\text{i}=1}^{\text{n}}\sum_{\text{j}=1}^{\text{n}}{\text{f}}_{\text{ij}}=\text{min}(\sum_{\text{i}=1}^{\text{n}}{\text{W}}_{\text{i}}^{\text{g}},\sum_{\text{j}=1}^{\text{n}}{\text{W}}_{\text{j}}^{\text{r}})$$

By adopting the Earth Mover's Distance (EMD) algorithm [18], which measures the least amount of work needed to match between two histograms through linear programming, a flow matrix **f** = {*f_ij_*} from one histogram to another can be obtained. The *f_ij_* is the pixel number transited from bin *i* in one histogram to bin *j* in another. Then, we propose a relative albedo function between the green and red channels as Equation (11) and obtain the estimated relative reflectance ratio *α̃*(i) for each grey level.

(11) $$\tilde{\alpha }(\text{i})=\frac{\left(\sum_{\text{j}=1}^{\text{n}}{\text{f}}_{\text{ij}}\right)\text{i}}{\sum_{\text{j}=1}^{\text{n}}\sum_{\text{j}=1}^{\text{n}}\left({\text{f}}_{\text{ij}}\text{j}\right)},1\leq \text{i}\leq \text{n},1\leq \text{j}\leq \text{n}$$

The relative reflectance ratio *β̃*(i) can be estimated in a similar manner. After calibration, *I_a_* serves as the input for stripe segmentation and color classification.

## 4. Stripe Segmentation and Peak Localization

After static and adaptive color calibration, the color of the stripe can be classified correctly in the following Section 5. However, the pixel-wise *O* and red channel reflectance ratio *a^r^* still remain in *I_a_*, making effective stripe segmentation and accurate peak localization challenging. Thus, we propose a novel *M* channel and DTT algorithm to derive robust stripe segmentation results.

### 4.1. M Channel Definition

An *M* channel, which is a function of the RGB channels in *I_a_*, is proposed in Equation (12) to suppress ambient light *O*.

(12) $${\text{M}}_{\text{ij}}=\text{max}({\text{C}}_{\text{ij}}^{\text{r}},{\text{C}}_{\text{ij}}^{\text{g}},{\text{C}}_{\text{ij}}^{\text{b}})-\text{min}({\text{C}}_{\text{ij}}^{\text{r}},{\text{C}}_{\text{ij}}^{\text{g}},{\text{C}}_{\text{ij}}^{\text{b}})$$

where ${\text{C}}_{\text{ij}}^{\text{r}}$ in Equation (13) is the red channel value of pixel (*i, j*) in *I_a_*, and the superscripts r, g and b denote the red, green and blue channels respectively.

(13) $$\left(\begin{array}{c} {\text{C}}_{\text{ij}}^{\text{r}}\\ {\text{C}}_{\text{ij}}^{\text{g}}\\ {\text{C}}_{\text{ij}}^{\text{b}} \end{array}\right)={\text{a}}^{\text{r}}\left(\begin{array}{c} {\text{p}}_{\text{ij}}^{\text{r}}+\\ {\text{p}}_{\text{ij}}^{\text{g}}+\\ {\text{p}}_{\text{ij}}^{\text{b}}+ \end{array}\begin{array}{c} {\text{o}}_{\text{ij}}^{\text{r}}\\ {\text{o}}_{\text{ij}}^{\text{g}}\\ {\text{o}}_{\text{ij}}^{\text{b}} \end{array}\right)$$

Given the assumption that ambient light is mostly white light, *i.e., o^r^* ≈ *o^g^* ≈ *o^b^*, the *M* channel can be simplified as Equation (14).

(14) $$\begin{array}{cc} {\text{M}}_{\text{ij}} & ={\text{a}}^{\text{r}}\text{max}\left(\begin{array}{c} {\text{p}}_{\text{ij}}^{\text{r}}+\\ {\text{p}}_{\text{ij}}^{\text{g}}+\\ {\text{p}}_{\text{ij}}^{\text{b}}+ \end{array}\begin{array}{c} {\text{o}}_{\text{ij}}^{\text{r}}\\ {\text{o}}_{\text{ij}}^{\text{g}}\\ {\text{o}}_{\text{ij}}^{\text{b}} \end{array}\right)-{\text{a}}^{\text{r}}\text{min}\left(\begin{array}{c} {\text{p}}_{\text{ij}}^{\text{r}}+\\ {\text{p}}_{\text{ij}}^{\text{g}}+\\ {\text{p}}_{\text{ij}}^{\text{b}}+ \end{array}\begin{array}{c} {\text{o}}_{\text{ij}}^{\text{r}}\\ {\text{o}}_{\text{ij}}^{\text{g}}\\ {\text{o}}_{\text{ij}}^{\text{b}} \end{array}\right)\\ & ={\text{a}}^{\text{r}}\text{max}\left(\begin{array}{c} {\text{p}}_{\text{ij}}^{\text{r}}\\ {\text{p}}_{\text{ij}}^{\text{g}}\\ {\text{p}}_{\text{ij}}^{\text{b}} \end{array}\right)-{\text{a}}^{\text{r}}\text{min}\left(\begin{array}{c} {\text{p}}_{\text{ij}}^{\text{r}}\\ {\text{p}}_{\text{ij}}^{\text{g}}\\ {\text{p}}_{\text{ij}}^{\text{b}} \end{array}\right)\\ & ={\text{a}}^{\text{r}}\text{max}\left(\begin{array}{c} {\text{p}}_{\text{ij}}^{\text{r}}\\ {\text{p}}_{\text{ij}}^{\text{g}}\\ {\text{p}}_{\text{ij}}^{\text{b}} \end{array}\right) \end{array}$$

Note that there is at least one invalid color channel in the designed stripe patterns. Thus, $\text{min}({\text{p}}_{\text{ij}}^{\text{r}},{\text{p}}_{\text{ij}}^{\text{g}},{\text{p}}_{\text{ij}}^{\text{b}})$ is always zero. As a result, the *M* channel suppresses white ambient light. However, the red channel reflectance ratio *a^r^* is still an interference.

**Figure 2.**
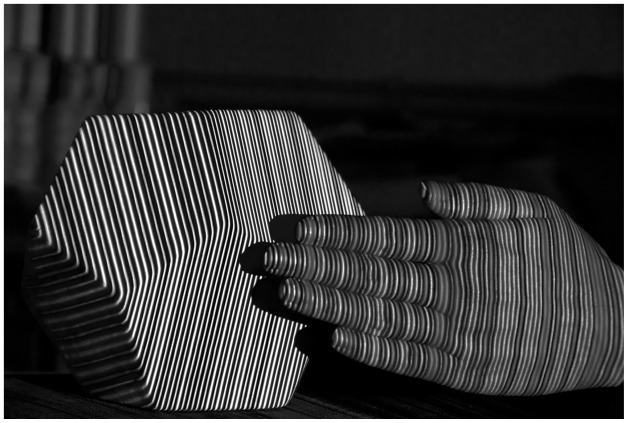
An example of *M* channel.

**Figure 3.**
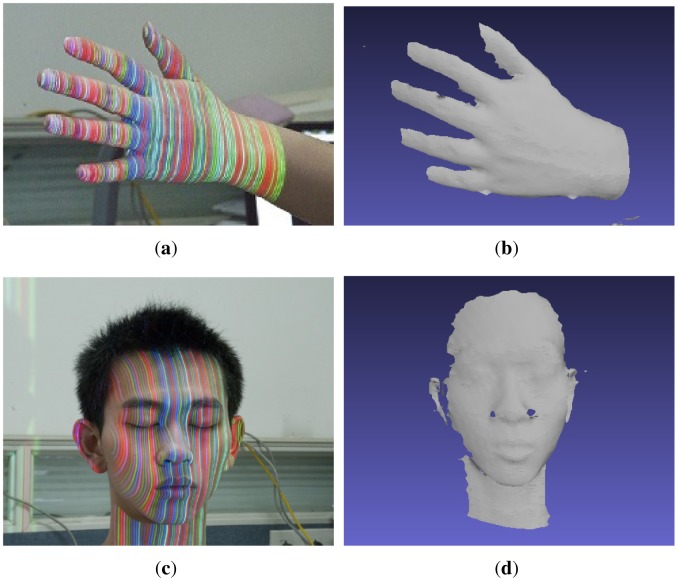

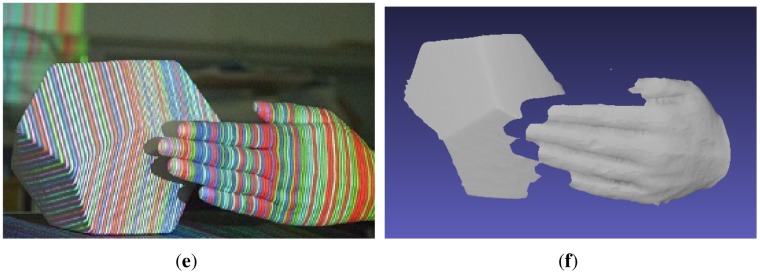
Reconstruction examples: (**a**) hand; (**c**) face and (**e**) hand with background of hexahedron under the pattern illumination. Reconstructed surface of the (**b**) hand; (**d**) face and (**f**) hand with background of hexahedron.

Another advantage of the *M* channel is that it integrates the RGB color channels into one channel while maintaining the original characters, which facilitates further processing. Figure 2 is the *M* channel of Figure 3(e).

### 4.2. Stripe Segmentation

The stripe pattern is vertical, and we process the *M* channel horizontally. A stripe in a captured image means a peak in the *M* channel. Instead of segmenting stripes by illuminance value, we detect stripes by locating the intensity rising and falling edges in the *M* channel. The rising edge and falling edge information is derived by applying DTT in Equations (15,16).

(15) $${\text{T}}_{\text{ij}}=\sum_{\text{k}=\text{h}+1}^{\text{j}+\text{N}}\sum_{\text{h}=\text{j}}^{\text{j}+\text{N}-1}\text{sign}({\text{M}}_{\text{ik}}-{\text{M}}_{\text{ih}})$$

and

(16) $$\text{sing}(\text{a})=\{\begin{array}{cc} 1 & \text{a}>0\\ 0 & \text{a}=0\\ -1 & \text{a}<0 \end{array}$$

where *T_ij_* indicates the local trend of pixel (*i, j*) in the M channel. The local trend measures the probability of an increasing or decreasing trend in a given window. The size of the window is controlled by *N*, which is typically assigned a value of less than half of the stripe width in the captured image.

In Equation (15), |*k* – *h*| ≤ *N*. Because *N* is a small number, we assume that the reflectance ratio is locally continuous, *i.e.*, ${\text{a}}_{\text{ik}}^{\text{r}}\approx {\text{a}}_{\text{ih}}^{\text{r}}$. Then, we obtain Equation (17).

(17) $$\begin{array}{cc} \text{sing}({\text{M}}_{\text{ik}}-{\text{M}}_{\text{ih}}) & =\text{sing}({\text{a}}_{\text{ik}}^{\text{r}}\text{max}\left(\begin{array}{c} {\text{p}}_{\text{ik}}^{\text{r}}\\ {\text{p}}_{\text{ik}}^{\text{g}}\\ {\text{p}}_{\text{ik}}^{\text{b}} \end{array}\right)-{\text{a}}_{\text{i},\text{h}}^{\text{r}}\text{max}\left(\begin{array}{c} {\text{p}}_{\text{ih}}^{\text{r}}\\ {\text{p}}_{\text{ih}}^{\text{g}}\\ {\text{p}}_{\text{ih}}^{\text{b}} \end{array}\right))\\ & =\text{sing}(\text{max}({\text{p}}_{\text{ik}})-\text{max}({\text{p}}_{\text{ih}})) \end{array}$$

where ${\text{p}}_{\text{ik}}={\left({\text{p}}_{\text{ik}}^{\text{r}},{\text{p}}_{\text{ik}}^{\text{g}},{\text{p}}_{\text{ik}}^{\text{b}}\right)}^{\text{T}}$ and ${\text{p}}_{\text{ih}}={\left({\text{p}}_{\text{ih}}^{\text{r}},{\text{p}}_{\text{ih}}^{\text{g}},{\text{p}}_{\text{ih}}^{\text{b}}\right)}^{\text{T}}$. Thus, Equation (15) can be rewritten as Equation (18). *T_ij_* is only related to the projected pattern and is independent of the reflectance ratio and ambient light.

(18) $${\text{T}}_{\text{ij}}=\sum_{\text{k}=\text{h}+1}^{\text{j}+\text{N}}\sum_{\text{h}=\text{j}}^{\text{j}+\text{N}-1}\text{sing}(\text{max}({\text{p}}_{\text{ik}})-\text{max}({\text{p}}_{\text{ih}}))$$

The DTT in Equation (18) works in the following manner:

- at the falling edge in the *M* channel, *T* arrives its maximum $\frac{\text{N}(\text{N}+1)}{2}.$
- at the rising edge in the *M* channel, *T* arrives its minimum $-\frac{\text{N}(\text{N}+1)}{2}.$
- otherwise, the value of *T* is between maximum and minimum.

**Figure 4.**
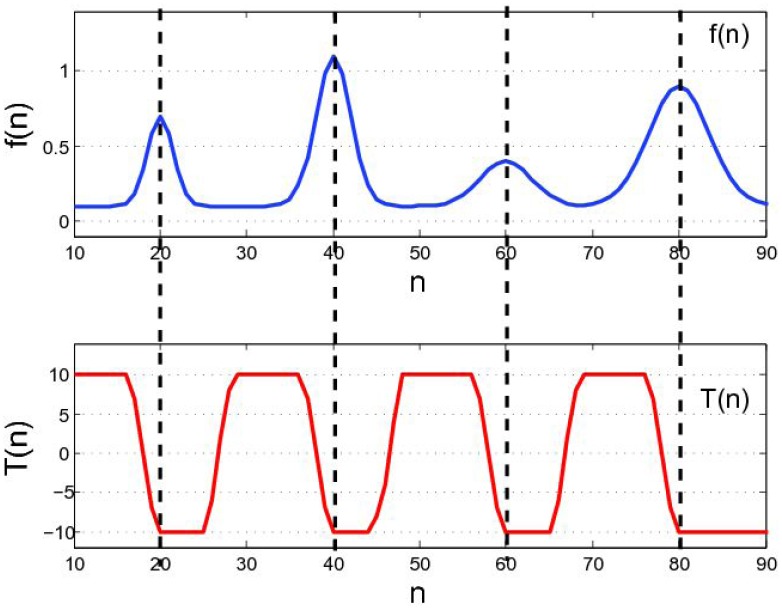
Robustness of local trend. Four peaks in *f*(*n*) conform to Gaussian distribution and have different arguments (stripe width and amplitude), but local trend *T* has the same max-to-min transition pattern at each peak position.

**Figure 5.**
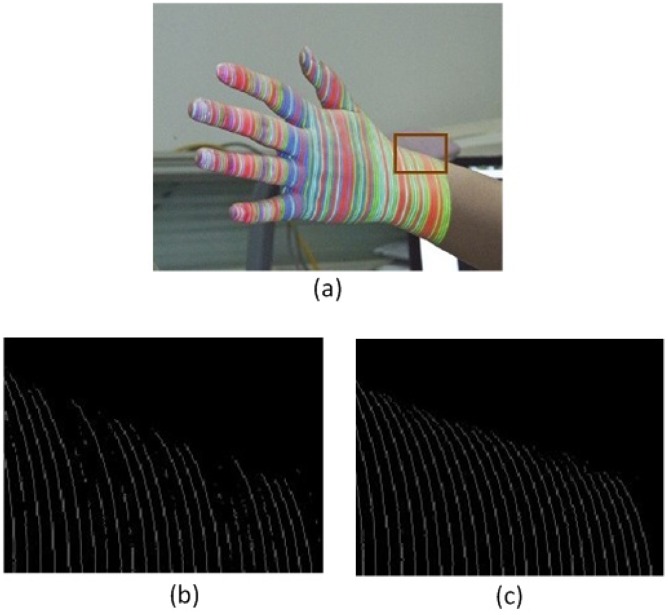
A comparison of stripe segmentation result. (**a**) captured source image; (**b**) stripe segmentation result using local adaptive thresholding method; (**c**) stripe segmentation result using DTT.

Figure 4 clarifies how DTT works. For example, *f*(*n*) is a scan-line in the *M* channel. If *f*(*n*) is locally monotonically decreasing in the window, then *T* achieves its maximum value $\frac{\text{N}(\text{N}+1)}{2}$. In contrast, *T* reaches its minimum value $-\frac{\text{N}(\text{N}+1)}{2}$ at *f*(*n*)'s rising edge. The values between the maximum and minimum denote that *f*(*n*) is neither monotonically increasing nor decreasing. The transition from the maximum to minimum values in *T* indicates a peak in the M channel. Thus, the max-to-min transition in *T* is searched for to locate the area of stripes. The rising edge in the M channel is marked as the start of a stripe area, and the falling edge in the M channel is marked as the end of a stripe area. The result of peak detection using DTT is illustrated in Figure 5. We can determine that DTT is more robust than the local adaptive threshold method [19] for weak stripes.

With the help of the *M* channel and DTT, there is no need to cut the foreground from the background. The standard of DTT detecting color stripes is a strict rising edge and strict falling edge in the *M* channel. The standard is so strict that very few points in the background are taken as candidate stripes after DTT. The robustness is enhanced by excluding the influence of the background.

### 4.3. Peak Localization

Accurate sub-pixel peak localization must be estimated to derive the 3D point cloud. Some existing sub-pixel peak localization algorithms are detailed and compared in [20]. To estimate the peak position accurately, the maximum value *M_max_* of the *M* channel is searched for in each stripe area, and its position is labeled as *I_max_*. The estimated sub-pixel peak position *I_estimated_* is proposed as Equation (19):

(19) $${\text{I}}_{\text{estimated}}=\frac{\sum {\text{I}}_{\text{i}}\cdot {\text{M}}_{\text{i}}}{\sum {\text{I}}_{\text{i}}},\text{for}{\text{M}}_{\text{i}}\geq \alpha {\text{M}}_{\text{max}}$$

where *I_i_* is the horizontal offset, *M_i_* is the *M* channel's intensity value, and *α* is the related ratio, which defines the pixels related to *M_max_* around *I_max_*. In the following experiments, *α* is set to 0.8.

## 5. Color Classification

The color is classified in HUV space. Assuming that ambient light is mostly white light and taking the color channel *C^r^* > *C^g^* > *C^b^* as an example, we calculate the hue value from Equation (20).

(20) $$\text{h}=\frac{{\text{C}}^{\text{g}}-{\text{C}}^{\text{b}}}{{\text{C}}^{\text{r}}-{\text{C}}^{\text{b}}}\cdot \frac{\pi }{3}=\frac{{\text{a}}^{\text{r}}({\text{p}}^{\text{g}}+{\text{o}}^{\text{g}})-{\text{a}}^{\text{r}}({\text{p}}^{\text{b}}+{\text{o}}^{\text{b}})}{{\text{a}}^{\text{r}}({\text{p}}^{\text{r}}+{\text{o}}^{\text{r}})-{\text{a}}^{\text{r}}({\text{p}}^{\text{b}}+{\text{o}}^{\text{b}})}\cdot \frac{\pi }{3}=\frac{{\text{p}}^{\text{g}}-{\text{p}}^{\text{b}}}{{\text{p}}^{\text{r}}-{\text{p}}^{\text{b}}}\cdot \frac{\pi }{3}$$

where (*C^r^, C^g^, C^b^*) is the color intensity in *I_a_*. In other cases, *h* can be calculated by equations similar to Equation (20). Equation (20) demonstrates that the *O* and *a^r^* are canceled out, and the *h* of the colors in *I_a_* is only related to the projected patterns. Thus, the classification process is independent of the reflectance characteristics of the scanned objects and white ambient light.

The correspondence between the detected and projected patterns was established by applying dynamic programming [16]. We compare three detected neighboring stripes that each have three projected neighboring stripes. The sum of the hue value difference is defined as the score function. By considering neighboring conditions, we can accurately handle the edge conditions when occlusion occurs.

## 6. Experiments and Discussions

### 6.1. Adaptive Color Calibration Performance

**Figure 6.**
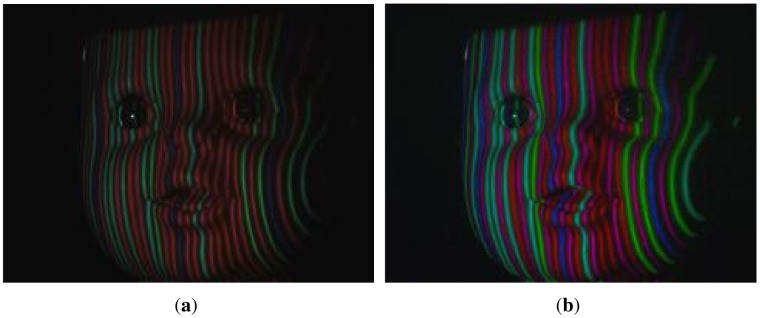

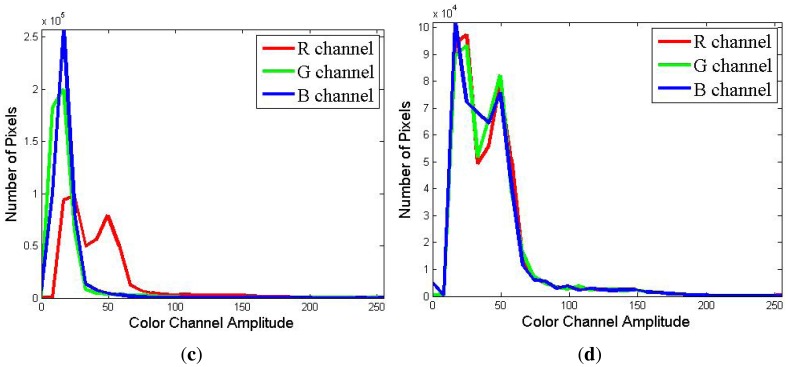
An example of adaptive albedo calibration. (**a**) face model under illumination patterns; (**b**) calibrated image of face model; (**c,d**) histograms of (a,b) respectively.

**Figure 7.**
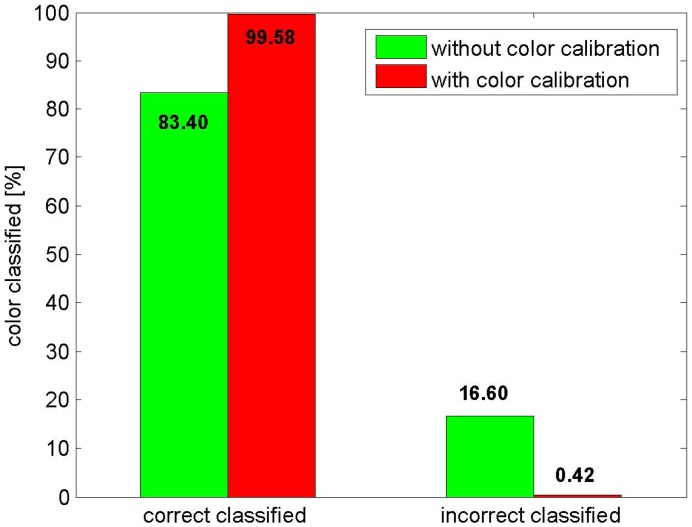
A comparison of color classification results.

Adaptive color calibration is a crucial method for assuring the accuracy rate of color classification. This method can adjust a stripe's color adaptively with respect to the different albedos in the RGB channels. Figure 6(a,c) illustrates that the gain of the R channel is stronger than those of the G and B channels. After adaptive color calibration, the gains of the three channels are almost same in Figure 6(b,d). Some quantitative comparisons between the adaptive albedo calibration and non-adaptive calibration have been performed. The results in Figure 7 clearly show that adaptive albedo calibration increases the number of correctly labeled points (by more than 15%).

### 6.2. Peak Localization Performance

In this subsection, we mainly focus on analyzing the error of the estimated sub-pixel peak position. The performance of the traditional methods is compared with our method, which is referred to as the Max-Min Weighted Average Method (**MMWA**). Some traditional methods are listed as follows:

- *Max Method* (**MAX**) [2]. Pages *et al.* use the maximum intensity value to define the M channel and choose localizations where the M channel reaches its maximum value as the estimated peak position.
  (21) $$\begin{array}{c} {\text{I}}_{\text{i}}=\text{max}({\text{R}}_{\text{i}},{\text{G}}_{\text{i}},{\text{B}}_{\text{i}})\\ \text{center}=\text{x}\;\text{where}\;{\text{I}}_{\text{x}}=\text{max}({\text{I}}_{\text{i}}) \end{array}$$
- *Weighted Average Method* (**WA**) [21]. This method calculates the average intensity value of the RGB channels and uses a weighted algorithm in the entire area of the stripe to derive the peak position.
- *Midpoint Method* (**MID**) [12]. This method simply uses the midpoint of the stripe as the feature point.
- *Probability Method* (**PM**) [15]. Fechteler *et al.* use the probability method to estimate the peak localization. They detect the maximum in each RGB channel and assign each color peak a probability of being a valid stripe. Peak localization is estimated by these probabilities.
  (22) $$\text{center}=\frac{{\text{C}}_{1}*{\text{P}}_{1}+{\text{C}}_{2}*{\text{P}}_{2}}{{\text{P}}_{1}+{\text{P}}_{2}}$$
  where *P_i_* is the probability of *C_i_* being a valid stripe

To simulate the captured stripes, we generate a designed color stripe pattern. As shown in Equation (23), the intensity of a valid color channel is consistent with the Gaussian Distribution and corrupted with noise. Each RGB channel is additionally polluted by some offset *o* simulating ambient light. For example, a red stripe compromises a valid red channel generated by Equation (23), while invalid Green and Blue channels contain offset *o* only.

(23) $$\text{I}(\text{n},\text{c},\sigma ,\beta ,\text{A},\text{o})={\text{Ae}}^{-\frac{{\left(\text{n}-\text{c}\right)}^{2}}{2\sigma^{2}}}+\beta \text{A}ɛ+\text{o}$$

where *A* denotes the amplitude of the color intensity, *n* is the measured pixel, *βA* is the noise amplitude and *ε* ∈ (0, 1). We consider different noise levels: SNR = 25 dB, 20 dB and 18 dB. *c* is the stripe peak position, *σ* controls the stripe width and *o* is the intensity offset.

The RMS error of peak localization *c*, defined as Equation (24), is measured in each method by analyzing 10,000 samples. The average RMS error at the different noise levels are listed in Table 2, as *σ* changes from 0.3 to 0.6.

(24) $$\text{RMS}\_\text{Error}=\sqrt{\frac{\sum {\left({\text{c}}_{\text{i}}-{\text{c}}_{\text{i}}^{\prime }\right)}^{2}}{\text{N}}}$$

**Figure 8.**
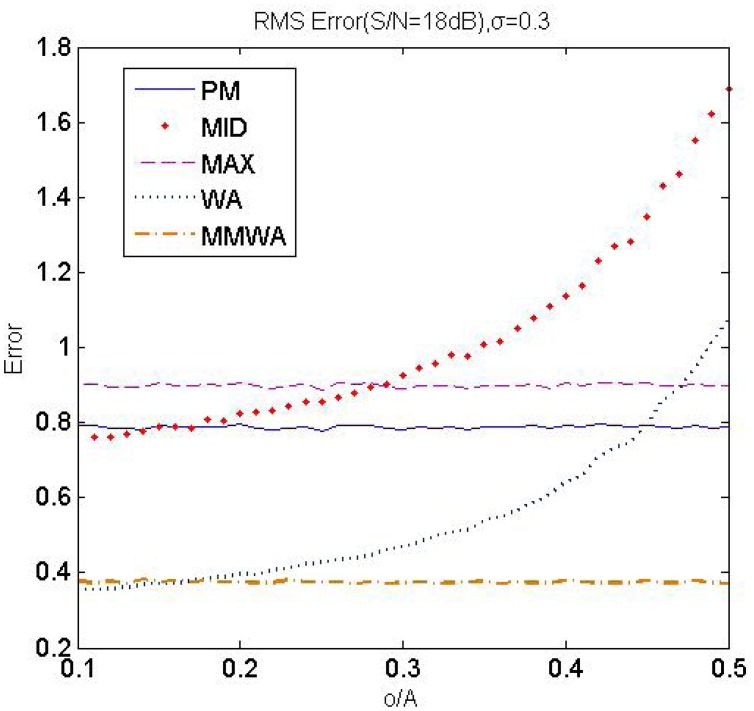
RMS error versus o/A (SNR = 18 dB,*σ* = 0.3).

In all of the methods, the RMS error increases as the noise increases from 25 *dB* to 18 *dB*, while **MMWA** derives the least RMS error at the same noise conditions. Figure 8 depicts that **WA** and **MID** are sensitive to light offsets. Nevertheless, our method obtains a high level of accuracy, even in a strong ambient light environment, because the offset is suppressed when deriving the M channel. Form Table 2 and Figure 8, we can conclude that our method outperforms other methods in terms of RMS error with respect to noise, stripe width and ambient light.

### 6.3. Reconstruction Results

Figure 3(a,c) demonstrates that the hand and face are projected with stripe patterns in a natural environment. With the presented methods, the color of the stripes could be classified correctly, the weak stripes could be detected effectively and the surfaces of the object could be reconstructed robustly, even when illuminated by such a strong ambient light, as depicted in Figure 3(b,d). Meanwhile, the reconstructed surfaces of the hand and face illustrate the richness in detail. Figure 3(b,d) contains 85,687 and 75,371 vertices respectively. Note that there is a hole near the nose in Figure 3(d), due to the occlusion in the captured image. Furthermore, Figure 3(e,f) demonstrates that the 3D shapes of multiple objects with different albedos in the same scene could be reconstructed simultaneously because color was calibrated adaptively according to the gray level and the stripes were segmented by DTT.

## 7. Conclusions

We have presented a color structured light system for robust 3D shape acquisition with regard to the reflectance characteristics of the scanned object, ambiguity due to uneven surfaces and white ambient light. Our contributions lie in two aspects of the proposed approach. First, we proposed a novel method for calibrating color adaptively according to the colored objects and white ambient light in the scene, as Sections 3.2 and 3.3 discussed, thus enhancing the robustness of the system and widening the range of potential applications. The second contribution lies in the effectiveness with which weak stripes caused by uneven surfaces of the scanned object can be found, by using a M channel and a DTT algorithm. Furthermore, we proposed an algorithm to locate the sub-pixel peaks of stripes. Through some experimental evaluations, we demonstrated that this structured light system employing the proposed techniques could obtain high-resolution 3D reconstructions without the need of dark laboratory environments.

## Figures and Tables

**Table 1.** Color assignment for each element.

**Element**	**Color**(*R, G, B*)	**Hue Value**
0	*red*(255, 0, 0)	0°
1	*green*(0, 255, 0)	60°
2	*blue*(0, 0, 255)	120°
3	*cyan*(0, 255, 255)	180°
4	*magenta*(255, 0, 255)	240°
5	*yellow*(255, 255, 0)	300°

**Table 2.** Average RMS Error at different noise.

**SNR**	**MMWA**	**PM**	**MID**	**MAX**	**WA**
25 dB	0.27	0.75	0.89	0.87	0.38
20 dB	0.46	1.05	1.04	1.14	0.54
18 dB	0.62	1.21	1.12	1.28	0.61
